# Talk, Text, Tag? Understanding Self-Annotation of Smart Home Data from a User’s Perspective

**DOI:** 10.3390/s18072365

**Published:** 2018-07-20

**Authors:** Emma L. Tonkin, Alison Burrows, Przemysław R. Woznowski, Pawel Laskowski, Kristina Y. Yordanova, Niall Twomey, Ian J. Craddock

**Affiliations:** 1 Faculty of Engineering, University of Bristol, Bristol BS8 1UB, UK; alison.burrows@bristol.ac.uk (A.B.); p.r.woznowski@bristol.ac.uk (P.R.W.); laskowski.pwl@gmail.com (P.L.); Kristina.yordanova@uni-rostock.de (K.Y.Y.); niall.twomey@bristol.ac.uk (N.T.); ian.craddock@bristol.ac.uk (I.J.C.); 2 Institute of Computer Science, University of Rostock, 18059 Rostock, Germany

**Keywords:** ground-truth acquisition, self-annotation, labelling, activity logging, location, NFC, smart homes, naturalistic data

## Abstract

Delivering effortless interactions and appropriate interventions through pervasive systems requires making sense of multiple streams of sensor data. This is particularly challenging when these concern people’s natural behaviours in the real world. This paper takes a multidisciplinary perspective of annotation and draws on an exploratory study of 12 people, who were encouraged to use a multi-modal annotation app while living in a prototype smart home. Analysis of the app usage data and of semi-structured interviews with the participants revealed strengths and limitations regarding self-annotation in a naturalistic context. Handing control of the annotation process to research participants enabled them to reason about their own data, while generating accounts that were appropriate and acceptable to them. Self-annotation provided participants an opportunity to reflect on themselves and their routines, but it was also a means to express themselves freely and sometimes even a backchannel to communicate playfully with the researchers. However, self-annotation may not be an effective way to capture accurate start and finish times for activities, or location associated with activity information. This paper offers new insights and recommendations for the design of self-annotation tools for deployment in the real world.

## 1. Introduction

The rise in the cost of health and social care is an increasingly familiar challenge, which has been attributed in part to an ageing population [1]. This means that many people are living longer, often with one or more long-term health conditions. Pervasive technologies have been proposed as one way to address this problem, as these technologies raise the possibility of supporting people through various stages of their lives [2,3]. Sensor-backed health monitoring has become a commercial reality in various contexts such as wearable devices [4], a range of which are available on the market and are widely used. The use of sensors, including within the home environment [5,6], has the potential to empower people to better understand their own health and wellbeing. This has fuelled a vision of ‘smart environments’ capable of supporting the prompt delivery of appropriate services in various domains, such as health and social care [5,7,8], by identifying the state of the environment, its occupants, and intervening in order to optimise both [9]. For example, for those recovering from a medical intervention, it could provide information to make decisions about their ongoing care; for those with diagnosed conditions, it could be useful in managing their condition appropriately; for others, it could support diagnosis, or underpin decisions related to improving lifestyle. However, in general, successful implementation of such a system requires the ability to relate sensor data to human behaviour—to identify activities within the data and to correctly label them. This labelling is referred to as ‘*annotation*’ of data [10].

Labelling sensor data can be done either online or offline. In the first case, the annotation is produced in parallel with the collection of data [11]. In the second case, the data is usually recorded together with a video of the observed activities, which allows the labelling to be performed subsequently based on the video log [12]. In both online and offline annotation, there are different practices, ranging from manual to semi-automatic and automatic approaches. Online manual approaches rely on an annotator who is present during the data collection and who observes the trial participants to label their behaviour [13,14]. Another option is to let the research participants annotate their own activities [15]. Offline manual approaches usually rely on the video log to create an annotation of the observed behaviour [12]. In contrast to manual approaches, semi-automated and automated approaches rely on a part of the data that is manually annotated to train a model. This model is then used to either suggest labels when new data is present or to automatically annotate the remaining data [16].

This paper focuses on manual approaches to online annotation, where the research participants annotate their own data. This is in line with calls for mechanisms that allow people to reason about their own data, with a view to producing situated accounts that are appropriate and acceptable [17,18]. The research presented here was conducted within a prototype smart home, which offered a unique opportunity to test various live annotation approaches with participants living in a naturalistic environment and performing unscripted activities as they would in their own homes. To this end, participants used an app with four available annotation modalities—voice, NFC (Near Field Communication), room-based list and manual entry using a touchscreen [15]. This paper is therefore likely to be relevant to those seeking to use similar self-annotation and ‘in-the-wild’ approaches. By drawing on the diverse expertise of the authors—which includes human–computer interaction (HCI), machine learning, system design and knowledge and information management—we acknowledge that annotation is a multidisciplinary problem of relevance to several fields, as has been previously noted [10]. Accordingly, we present both quantitative and qualitative data and analyses related to the use of the self-annotation app. While pertinent to this topic, it is beyond the scope of this paper to include the sensor data, which will be the focus of further work reported elsewhere.

We begin in Section 2 with a review of existing systems used for annotation in various contexts and disciplines, as well as discussing considerations for self-annotation. Section 3 provides an overview of the annotation app and how it was used in our study, to produce the results reported in Section 4. Specifically, this comprises findings from 12 participants, including data generated through each annotation modality and through post-stay semi-structured interviews. We discuss these findings in Section 5, in light of relevant theory and related work. By drawing on this evidence, we provide recommendations to guide the design of future annotation tools. The paper concludes with Section 6, which summarises this work and provides directions for future research. This paper extends on the preliminary work described in [15], by presenting the results of the study in full and offering additional insights into self-annotation, the use of various modes for annotation, and annotation in naturalistic contexts. The main focus of our earlier paper was to describe the rationale and evolution of the self-annotation app, although we also discussed some results emerging from interviews with the first three study participants. This paper, on the other hand, presents an analysis of both interview and annotation data contributed by 12 people who took part in the study. Another unique aspect of this paper is the drawing together of knowledge from a number of fields, which we review in the following section.

## 2. Related Work

Whilst annotation is essentially a classification task, it is a multidisciplinary research area that has been addressed differently according to discipline and context. Here, we draw together relevant work from various fields such as ubiquitous computing, HCI, psychology, and information sciences, among others.

### 2.1. The Role of Annotations in Lifelong Machine Learning

Automated and accurate activity recognition is a complex challenge that remains largely unsolved [19]. A central assumption in the development of smart environments is that human activity data generated by pervasive systems can be interpreted, and that the reliability of this interpretation is sufficiently high that the resulting information may be used as a basis for action. To validate this assumption, relevant sample data that has been labelled by one or more human experts is required. Acquiring this ground-truth can be reasonably straightforward in controlled environments such as laboratories [20,21,22,23]. Several critiques may be levelled at this approach to the problem. A principal limitation is that these approaches are not scalable and, therefore, hold limited practical value for real world deployments. The relevance of the data set to real world deployment scenarios may be limited due to the restricted context of collection [24]. In particular, participants may modify their behaviour and activities in response to the controlled environment, thus meaning that activity data, especially that relating to more complex activities, does not reflect natural human behaviour in other environments.

In recent years, the research community in pervasive computing has made a concerted effort to obtain a rich picture of natural human behaviour in real-life settings. A recent activity recognition challenge [13] introduced a new semi-naturalistic data set with several interesting features. Firstly, the data sequences were annotated by several annotators. Interestingly, this demonstrates the presence of annotation ambiguity on activity recognition data sets both in terms of the temporal alignment of the labels and the specification of the activities. Indeed, the regions of highest ambiguity are those with the highest rates of activity transitions. Since the labels themselves are ambiguous, evaluation of performance likewise becomes ambiguous in this setting. To overcome these difficulties, performance evaluation was based on proper measures between probability distributions. Several other data sets capture data and annotations in less controlled settings, but do not yet capture the aspects of activity required to be considered naturalistic. Additionally, related work [19] has concluded that since most activity recognition data sets have been collected in controlled lab environments, it is difficult to estimate performance of these methods in the wild. Therefore, there is a pressing need for naturalistic data sets, but several challenges are impeding the collection and release of naturalistic activity recognition data sets.

Recent applied research in activity recognition has highlighted a key challenge in the field of autonomous activity recognition: resolving the statistical differences in data collected in the lab and data collected in the wild [19]. This is seen in several recent works but can be summarised by a drop in predictive accuracy in the range of 30% to 50%. A well-accepted explanation of this deficit is that lab-based data often arises from well-controlled settings whereas home-based data are, by definition, naturalistic, chaotic and rich in variation. A formal methodology for bridging the performance gap is called domain adaptation [25] and is an active and ongoing area of research. An elegant modelling choice is to utilise transfer learning (a form of domain adaptation) in this setting, i.e., acquire a lab-based activity model but adapt its parameters to maximise expected performance in home settings. Even when activity models are successfully transferred, however, there are still some drawbacks with its deployment in longitudinal campaigns, most particularly the fact that the transferred model is static and will not adapt to emerging behavioural patterns. Therefore, a more compelling approach is to posit a model that both transfers into the new domain and can adapt to new data and annotations. Such a technique will not only be immediately deployable (overcoming the ‘cold start’ problem [26,27]) but speedily specialise to the layout of the new home [28,29] and can facilitate personalisation of models to individual users sequentially [30] (ultimately increasing their utility).

Although the model class described above constitutes an elegant and adaptive framework, a continuous stream of annotations will be required for optimal performance in general [31]. Thus, this solution is empowered only through the success and reliability of annotation acquisition, and this in turn relies on solutions to several considerations: bridging the lab/home gap, facilitating everyday annotation and self-annotation, understanding the language of annotation.

### 2.2. Other Forms of Annotation in Everyday Life

There are many reasons people annotate in everyday life. One such reason is to support user memory, as a cognitive aid. Lifelogging, for example, makes use of continuous documentation of aspects of everyday life in order to support and improve memory [32]. This purpose benefits significantly from encoding of relevant context—indexing of material is key, so lifelogging research calls for the collection of information such as spatiotemporal context, acoustic encoding and environmental data, in combination with automated or manual ‘tagging’ with metadata [32]. Additional data sources may also be considered, such as recording online activities [33]. Annotation, then, intrudes into lifelogging both directly as a datasource and for the purpose of validation of lifelogging technologies [34].

Another common reason for annotation is for purposes of personal information management, to support the curation of information objects or to provide support for ongoing tasks [35]. Personal information management (PIM) data sets have sometimes been used as part of an annotation strategy; for example, beyond its intended purpose, instant messaging may also contain useful information about location and activity [36]. As a research area, PIM is fragmented across several disciplines. Jones [37] identifies database management, information retrieval, information science, HCI, cognitive psychology and artificial intelligence amongst relevant subject areas; lifelogging might be said to be amongst them. PIM is ordinarily conceptualised as goal-centric; people’s aim is to manage, maintain, and when necessary retrieve the information that they require from their existing store of data—adding to it when they retrieve an additional salient data item from another source. However, some implementations and theoretical approaches to PIM do approach the types of problem more commonly associated with lifelogging—for example, ‘what was the website that Mary showed me last week?’ [37], or the broader conceptualisation of a digital ‘memory’ as something that we might review for the sake of interest or for fun.

A review of PIM literature shows that information is better recalled when stored in the same context in which it was learned [35], so PIM taxonomies benefit from a subjective, or personalised, aspect. Additionally, a finding of personal information requirement research of particular relevance to the development of annotation systems is that working in a general information management environment imposes a significant burden on the user, in part as a result of the need for users to ‘acquaint themselves with [the] classification system’ [35] (p. 9). For example, a process that requires a user to select terminology that is not familiar to them might require them to navigate a hierarchy of unfamiliar terms and consider each one to identify which term they consider most relevant to the concept they intend to express; such a process involves both cognition and storage of candidate terms and their locations in working memory. Hence, the design of classification systems requires a strong user-driven focus to minimise this gap. Personal classification systems are commonplace de facto approaches to working with information systems in general, often with a task-based focus (i.e., users tend to classify information according to perception of task) [38]. One flaw with this approach is the fragmentation that occurs, both between user approaches and within a given user’s data store, as the user moves from one task to another. The classical examples of such variation can be seen in ‘social tagging’ systems [39], where individual user metadata connected to resources is shared with other users, producing a widely variable set of annotations, some with a personal focus and some more broadly applicable.

### 2.3. Considerations about Self-Annotating Activity Data

In a study conducted by Tapia et al. [40], participants were given a device that prompted them every 15 min with questions about what they were doing, for how long they had been doing it, and whether they had been doing another activity before hand. The results of this annotation process lacked accuracy for a number of reasons—some activities were recorded by mistake, activities of short duration were difficult to capture, there were delays between the sensor firings and the labels of activities, fewer labels were collected than anticipated (low compliance), and sometimes participants specified one activity and carried out a different one. Some insights into these outcomes may be gleaned from the classical HCI problem of ensuring that a user completes all parts of a set task, as often exemplified by automatic teller machine (ATM) design. The technical requirements of building a secure ATM required system designers to add elements to the interaction flow that are incidental to the user’s perception of the goal [41], notably the need to enter a bank card, key in a PIN and retrieve the bank card. The user’s goal in this instance is to withdraw cash; the requirement to retrieve the bank card does not form part of this immediate goal. Users were so frequently observed leaving their bank cards in the ATM after receiving the cash that ATMs are now commonly designed to return the card before dispensing cash, hence placing this postcompletion task onto the critical path for completing the user goal [41,42]. The ATM design problem demonstrates ‘postcompletion error’, a type of error of omission that occurs as a result of user perception that the goal has been completed.

The primary goal of an individual self-annotating their activity is rarely annotation. Hence, the need to annotate the completion of a process is a ‘hanging postcompletion action’ [43]. The incidence of postcompletion errors relates to goal length and load placed on working memory [44]—that is, goal loss from working memory. Increase in complexity of the task may increase demand on working memory and, correspondingly, the rate of postcompletion errors [44]. We might therefore expect that annotators performing lengthier and more complex tasks are more likely to neglect postcompletion annotations.

Self-annotation constitutes an interruption of one or more activities undertaken towards fulfillment of a goal. Each annotation represents an interruption, and each is electively chosen by the self-annotating participant. This self-interruption—task-switching from their primary task to self-annotation at the participant’s own discretion—may be viewed as a form of discretionary task interleaving [45]. Tasks that are ‘internally interrupted’, i.e., interrupted at the user’s own discretion, are less likely to be resumed than tasks that are externally interrupted [45,46]. Furthermore, each interruption comes at a cost, since returning to a task requires the cognitively demanding process of reloading the task context into working memory [46]; representations present in working memory during the interruption may decay. Self-annotation through self-interruption can therefore incur a significant cost to the user, both cognitively and in terms of time taken. Cutrell et al. [47] suggest that participants may thus elect to delay switching between tasks until the completion of a subtask, i.e., a convenient time. This in itself may limit the quantity of annotations contributed by participants.

### 2.4. Choosing Labels

Interfaces are typically designed against an (abstract or concrete) understanding of the needs of the user community, including label-driven interfaces, which typically seek to follow the recommendation made by Nielsen [48] to ‘speak the user’s language’. Knowledge structures are ordinarily designed with a use case in mind [49]. However, user accessibility and familiarity are not primary factors in the development of most knowledge representation artefacts (concept labels in ontologies or subject headings in taxonomies). Knowledge structures may be used to support information management and querying tasks in such a way that the structure itself is not directly visible to the user [50]. The requirements of formal ontology differ sufficiently from user needs to render a level of abstraction beneficial [51]—that is, the information structure or visualisation a user sees may differ significantly from the internal knowledge representation (graph) on which the system depends.

A primary purpose of such abstraction is to resolve the issues that result from a lack of alignment between the knowledge representation system and the user’s ‘language’—conception of the domain, vocabulary use, etc. In particular, annotation tools designed for input and sometimes review of activity and context data are intended to support task-focused cognition, such as characterisation of activity and context, and data input, appropriate and consistent encoding of this information [52]. Tools that do not effectively support cognition may in general be expected to increase cognitive load. In general, annotation tasks impose high cognitive load [53]. In particular, prototype theory [54] suggests that more ambiguous exemplars are more difficult to categorise—the task of deciding whether such an exemplar should be characterised in a given category takes longer and achieves a lower level of inter-rater reliability. For example, if a cup of tea is clearly a hot drink, is a bowl of soup or noodles in broth categorised similarly, or does it belong elsewhere in a taxonomy of food? Such ambiguities are relatively time-consuming to resolve and may require navigation through a hierarchical ontology, hence imposing additional load on working memory. By providing abstractions that are more accessible to the user, some elements of this problem are mitigated. An alternative strategy is to minimise constraint placed on the user, although this may result in data that is more difficult to analyse.

A further complication is the fact that vocabulary use varies within and between populations. A commonly-identified example of this is the variation in terminology employed to describe particular meals. In Britain, for example, nomenclature for the afternoon and evening meals varies significantly within the population across multiple demographics, including age, location and social class. One person’s midday meal is ‘*lunch*’ while another’s is ‘*dinner*’. One person’s evening meal is ‘*dinner*’; another describes it as ‘*supper*’ or ‘*tea*’ [55]. In addition, such ambiguities are not confined to Britain or to the English language, with Rodríguez González et al. [55] identifying a similar effect in French and Spanish. Ambiguities of this kind are likely to be visible in free-text corpora. Unless given guidance to the contrary or constrained by the imposition of a controlled vocabulary, which may have implications in terms of cognitive load, annotations written by individuals are likely to reflect dialect and idiolect, i.e., the language habits of the group and of the individual [56]. The use of taxonomies (i.e., lists of terms) in user interface design may ‘mask’ ambiguities of this nature—that is, valid terms are used at each point, but inconsistencies may nonetheless exist in participant interpretations of these terms and emerge in later data analysis. In fact, researchers working with sensor data sets from various projects noted that, although they all contained activities with similar connotations, each used slightly different labels [57]. Those researchers also suggested that differences would also occur across the projects in the data sequences corresponding to similar labels, owing to subjective interpretation of the activities by the annotators.

Although term abstraction is considered an enabler of compatibility for users, the richness associated with such abstraction can also introduce ambiguity at several levels. Let us consider an application of automated activity classification in a smart home setting where labels are acquired in situ and the key objective is to learn a mapping from sensor data to predictions of ADLs (Activities of Daily Living). We have already seen how the definitions of ‘*dinner*’ and ‘*tea*’ can depend on context, and, critically, therefore, we must also be wary of their treatment in classification settings. In spite of the popularity of supervised machine learning, it is a paradigm that is not entirely compatible with the scenario described here due to label uncertainty. Instead, we can adopt methods from the field of weakly supervised learning [58]; a paradigm with relaxations on the requirements on label reliability and availability. Thus, by compensating for annotation inaccuracies with weakly supervised models, viable and consistent models can be produced that benefit directly from the practicalities of term abstraction.

## 3. Materials and Methods

**Figure 1:**
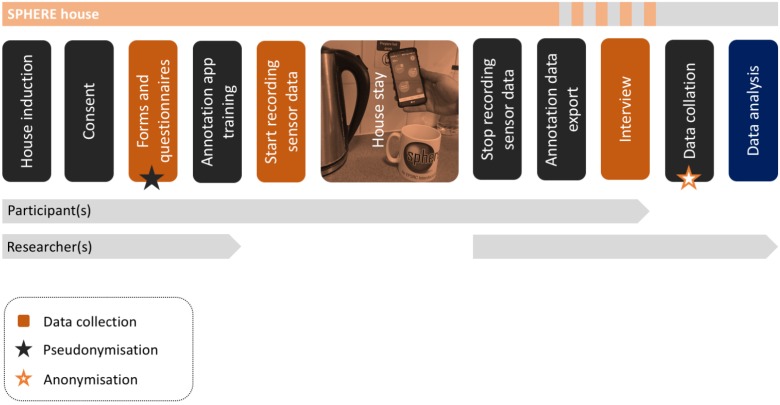
Study setup.

This sub-study was embedded within a larger interdisciplinary study, in which people were invited to live in a prototype smart home and were encouraged to live and behave as they do at home. Given this naturalistic approach, this exploratory sub-study aimed to understand people’s preferences for self-annotation, including but not limited to frequency of logging, type of activities logged, and preferred mode of logging. The study setup is depicted in Figure 1.

### 3.1. Setting and Sample

**Figure 2:**
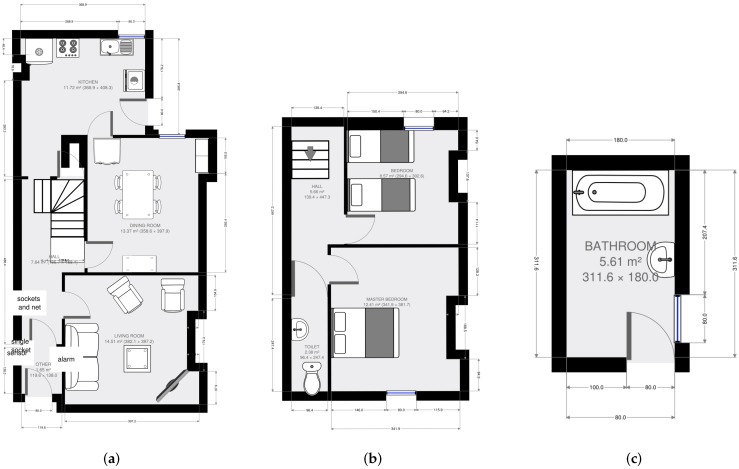
Floor plan of the prototype ‘*SPHERE house*’. A staircase joins (**a**) the ground floor to (**b**) the second floor; with (**c**) the bathroom half-way up.

Data collection took place in a prototype SPHERE house in Bristol (UK), between July 2016 and February 2018. The prototype SPHERE house is a residential property (the floorplan of which is shown in Figure 2), with a living room (Figure 2a), a study/dining room (Figure 2a), a kitchen (Figure 2a), a bathroom (Figure 2c) with a separate toilet (Figure 2b), two bedrooms (Figure 2b) and a small enclosed garden. This house has been fitted with the SPHERE system, which includes environmental, video and wearable sensors—full details of the platform described in detail in [6,59]. The sample for this study comprised 12 people (seven women), with ages ranging from 16 to 58 (median age: 25). Of these, three were not native English speakers. Four participants stayed for two nights and the remaining eight participants stayed for three nights. Six of the 12 participants were partners or friends who stayed concurrently, which means there were a total of nine individual or group stays for this data set.

### 3.2. Data Collection

At the start of their stay, participants were provided with the self-annotation app described in [15], also used during the 1st International Workshop on Annotation of useR Data for UbiquitOUs Systems (ARDUOUS) annotation session [10]. This app was designed to run on Android only and participants were given the option to use their own smartphones, provided the operating system was compatible, or to use the project’s smartphone for this purpose. It was explained to participants how to use the self-annotation app and they were asked to record activities using their preferred mode—the room-based list, voice, or NFC tags.

**Figure 3:**
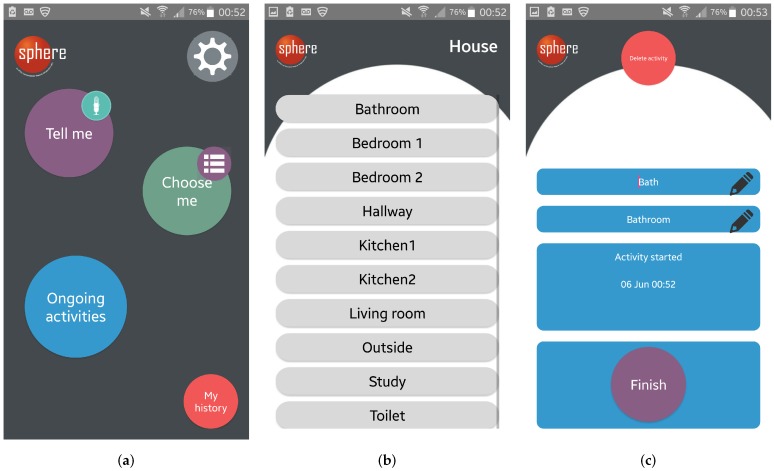
Screenshots of the ground-truth hybrid app. (**a**) main screen; (**b**) ‘*choose me*’ screen; (**c**) an ‘*ongoing activity*’ item.

To begin logging an activity, users could simply scan an NFC tag and the app would open automatically displaying a confirmation message; alternatively, from the main screen of the app (Figure 3a), users could select room-based logging (‘*Choose me*’) and select from a list of activities pre-defined for each room (Figure 3b), or voice-based logging (‘*Tell me*’) to describe activities freely through speech-to-text. The app afforded the option to manually edit any entry and to create additional activities under each location. All activities, irrespective of mode of logging, could be viewed in the ‘*Ongoing activities*’ screen where users could select an item from the list, edit its details, delete it or terminate it (Figure 3c). Users could also terminate an activity by scanning NFC tag for that activity, or terminate all ongoing activities with a single button press if, for example, they were leaving the house. A summary of these features is provided in Table 1.

Semi-structured interviews were conducted at the end of or soon after each stay by Alison Burrows. These interviews were an optional part of the study and two participants elected to not take part in the interviews, owing to time constraints. Interview topics of relevance to this article included thoughts about participants’ favourite and least favourite aspects of staying in the SPHERE house, as well as more targeted questions about logging activities and using the annotation app.

### 3.3. Data Analysis

Pseudoanonymised data collected through the annotation app were stored in a MongoDB database and were subsequently anonymised by Alison Burrows; for instance, she removed MAC addresses for participants who used their own phones and replaced actual dates with ‘*Day 1*’, ‘*Day 2*’, and so forth. This two-step process was necessary to completely break the link between any identifiable information and the participants it concerned, some of whom were known to the researchers. Annotation data were successfully logged for 10 of the 12 participants, with two failed instances later being diagnosed as caused by a connectivity problem (one case) and by problems with the server’s availability (one case). However, given that those two participants took part in the interview and reported using the app extensively during the study, they were not excluded from the interview data set. For the purpose of data analysis, all logged activities were normalised to the SPHERE ADL ontology terms [60]. Analysis was initially performed on the raw JSON data, in order to produce various visualisations presented here. Further analysis was conducted in Microsoft Excel on the collated data from the CSV export from Mongo DB, with a focus on mode of logging, frequency of logging, type of activities logged, among others (refer to the Supplementary Materials note).

Ten interviews were audio-recorded and transcribed in full. The interview transcripts were anonymised and sections relevant to this article were collated in a Microsoft Excel spreadsheet. The principles and procedures of thematic analysis [61] were followed for these data, whereby transcripts were inductively coded in order to identify meaningful themes in the data. Themes emerging from this analysis provided explanation and deeper understanding of the annotation data, as well as insights into how self-annotation can be improved from a user experience perspective.

## 4. Results

This section presents key findings pertaining to usage of the app alongside findings from the interviews, in order to provide a richer understanding of the participants’ experiences of self-annotation and the contextual factors that shaped them.

### 4.1. Mode of Logging

A total of 677 activities was logged across 10 participants. Table 2 shows that the NFC and voice modes of logging were used less frequently than the room-based list. Of the three modes available to start logging activities, 75 (~11%) activities were initiated via NFC, 17 (~2.5%) were initiated via voice, and the remaining 585 (~86.5%) through the room-based list. Participants who expressed a preference for using the room-based list valued its ease of use, reliability, clear feedback (each button changes to green when selected, as shown in Figure 3b), and conceptual model. Some participants also mentioned that, once they got into the habit of using one mode of logging and knew it worked, they tended to stick with it. Two participants reported enjoying using a combination of two modes, depending on the context. P5 said ‘*I really liked that in the kitchen you could just scan the activity on and off*’ and used the list-based logging in other rooms. P12 found using voice useful to annotate activities while moving around (e.g., getting ready to leave the house) because this only required being within range of the phone rather than having to hold it to make a selection. Although P3 seemingly used all three modes available to start logging an activity, they said of their experience of using voice-based logging: ‘*I was looking for an “Off” switch, an “I’ve finished speaking now” switch to say “Send it off” but, because there wasn’t one of those, initially, I just expected some feedback when I stopped speaking. It didn’t come for a long time and I got impatient and, therefore, I thought it wasn’t working, so then I started pressing buttons around the phone to try to tell it I’d stopped speaking and that I think cancelled it.*’ P3 felt the NFC feedback was ambiguous and this gave them the feeling that it only worked occasionally; they also explained that the location and position of some of the NFC tags had caused problems, for example, when they put their phone down on the bedside table and it accidentally started logging sleep (this entry was deleted by the participant).

To stop logging activities, the room-based list remained the most popular option with a total of 402 (~59.4%) activities terminated this way. NFC was used for this purpose 40 (~5.9%) times, the ‘*Ongoing activities*’ list was used 196 (~29%) times, the ‘*Finish all*’ option was used 28 (~4.1%) times, and 11 (~1.6%) activities were unterminated. Of the four participants who used NFC to log the start of an activity, only three used this feature to terminate activities. There was a similar decrease from the number of activities started with NFC (75 instances) to the number of activities terminated with NFC (40 instances). This may, at least in part, be due to the existence of additional ways of terminating activities compared to modes of initiating them. This suggests that there is value in a cross-modal approach as well as providing a way to terminate all activities at once.

### 4.2. Logging Activities

Table 2 shows that the average number of activities logged per day was 24 across all participants, with the highest average number being 35 activities per day (P3) and the lowest average number being 14 activities per day (P9). Of the 10 participants who took part in the interviews, only three mentioned enjoying annotating their activities or perceiving personal benefits from doing it. In particular, they felt this process enabled them to reflect on themselves, their routines, and how they actually use their time. For example, P6 said: ‘*I always thought I’m completely rushed off my feet and looking at it I actually have more time on my hands than I thought. So I guess it makes you reflect and say are you actually being as productive as you think you are. And I am actually giving myself more time to relax than I thought I did, so in a way that’s a good thing.*’

In contrast, several participants intimated they did not like self-annotating during their stay. Reasons for this included aspects related to the usability of the app, but more predominantly aspects related to the actual process of annotating activities. Most participants said it was easy to forget to carry the phone with them but also to forget to record their activities, which for some was not a normal thing to do and ‘*was at the back of our mind a lot of the time*’ (P9). Participants reported struggling with the cognitive load of the task of annotating activities, such as delineating activities that do not have a clear beginning and end, setting boundaries between co-occurring activities (e.g., having the television on while working), handling short and spontaneous activities. To be able to self-annotate in real time, participants had to plan an activity, start recording the activity, do the activity as planned and stop recording the activity—yet natural behaviours are never this clear-cut.

**Figure 4:**
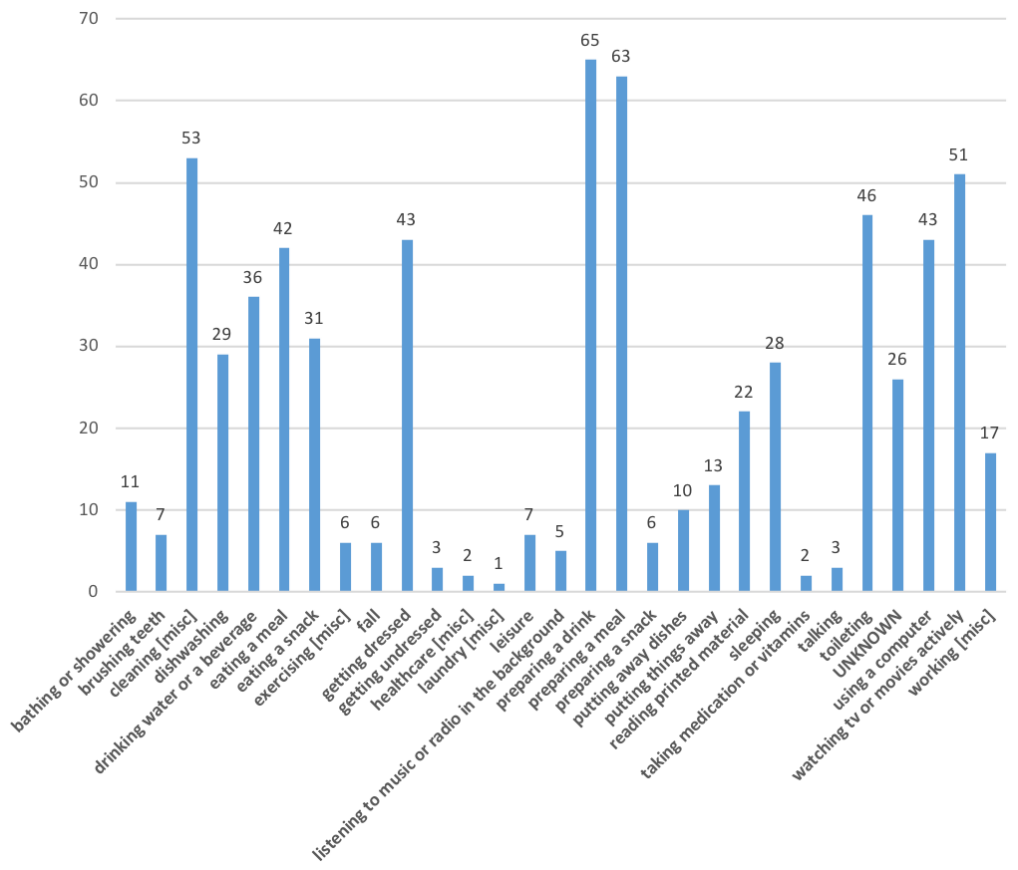
Total count of activities logged across all participants (normalised to ontology terms).

Figure 4 shows that frequently recorded activities tended to cluster around certain themes such as food (63 instances for ‘*preparing a meal*’, 42 instances for ‘*eating a meal*’, 31 instances for ‘*eating a snack*’) and drink (65 instances for ‘*preparing a drink*’, 36 instances for ‘*drinking water or beverage*’). These are activities that occur more regularly throughout the day than, for example, ‘*sleeping*’ (28 instances) and thus this relatively high number of logs is to be expected. Another way to interpret these results is that these are more clearly delineated activities when compared, for example, to ‘*talking*’ (three instances). The fact that certain activities were not recorded or were seldom recorded, such as one count for ‘*laundry*’, is likely due to the short-term nature of the stays.

#### 4.2.1. Distribution of Logged Terms

We employed a simple unigram-based co-occurrence model to inspect the most popular term co-occurrences. Prior to applying this, we began by normalising the terms provided by indexing each term against the SPHERE ontology [60,62]. Certain terms are already drawn from this ontology; others, however, are input by keyboard or voice and require mapping to ontology concepts. These terms have been manually aligned. Of particular interest to us were areas which we would ordinarily expect to exhibit a level of symmetry; for example, an individual might be expected to undress before sleeping as part of their getting ready for bed routine, and dress during their morning routine. Similarly, making a drink would ordinarily be expected to precede drinking a drink, and making food would ordinarily be expected to precede eating food.

**Figure 5:**
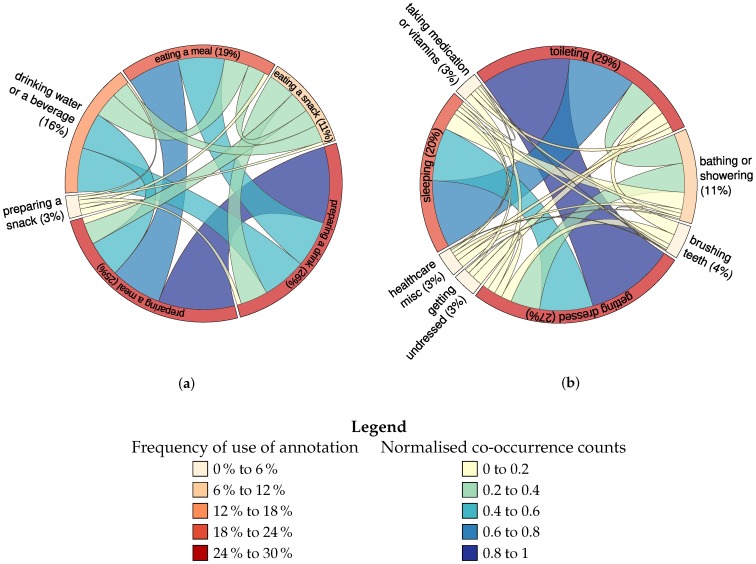
Distribution of term usage. (**a**) term co-occurrence in annotation data set for food and drink related vocabulary; (**b**) term co-occurrence in annotation data set for concepts and activities related to sleep and awakening.

**Figure 6:**
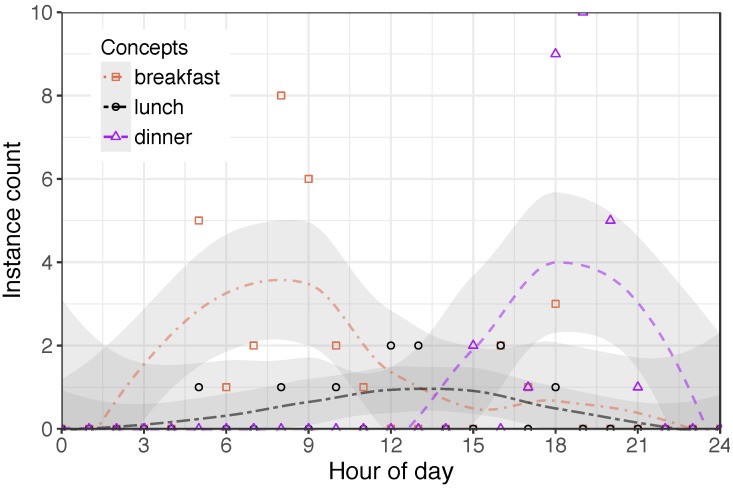
Terminology use in meal preparation as a function of the hour of day.

The chord diagram presented in Figure 5a displays frequency of use and co-occurrence counts for annotations relating to food and drink preparation and consumption. Figure 5b displays frequency of use and co-occurrence for annotations relating to sleep, preparation for sleep and waking. The labelled arc segments around the circle represent the frequency of use of annotations, whilst the size and colour-coding of the lines linking one arc segment to another represent the number of times these annotations co-occur: for example, ‘*toileting*’ and ‘*getting dressed*’ occur frequently alongside one another. Both co-occurrence and absolute counts are normalised for presentation purposes. This particular evaluation method deals with sequencing annotations rather than overlap time, unlike the analysis in Figure 6, which deals with overlap in term use throughout the day. Reviewing this shows that few ‘symmetrical’ actions co-occur. Preparing a snack is annotated much less frequently than eating a snack. Preparing a drink co-occurs somewhat weakly with drinking. Not only is preparing a drink annotated more frequently than drinking, but preparing a drink and drinking do not occur sequentially as often as, for example, preparing a drink and preparing a meal, or preparing a drink and eating a meal. A close inspection of the data suggests that participants seldom annotate both making a drink and drinking—most list only one or the other, but not both. However, participants often annotate both preparing a meal and eating the meal; meal preparation does occur more frequently than eating, but this may relate to participant preparation of meals that are not consumed within the home, such as sandwiches.

#### 4.2.2. Note on Daily Routines

One area of interest was to derive daily routines from the annotation log of a particular person. This was possible for tasks such as having breakfast, lunch and dinner (see Figure 6). However, we were unable to derive a complete routine including all repetitive tasks. There are several reasons for that. Firstly, given the various modes of logging and the possibility to edit entries, we found that participants used different terms to describe the same activity. While this could be expected between participants, it was also observed that a single person used varying terms to describe the same activity. For example, one participant used ‘*use device*’, ‘*using device*’, and ‘*use device (kitchen)*’ to describe the same activity. Another example is that, in addition to ‘*preparing breakfast/lunch/dinner*’, a participant used the label ‘*I’m cooking right now*’. Secondly, some activities could be described through different labels or one label belonging to a more general concept. In that case, people were unsure of which concept to use, or if they should decide on the broader concept. For example, a participant who stayed for three days in the SPHERE house had only one annotation of ‘*brush teeth*’. This could be explained by the fact that they then chose to use the broader concept ‘*personal hygiene*’ instead. Lastly, short actions were often not annotated, while longer actions were usually included in the annotation. For example, one participant annotated the preparation of a drink 13 times. There is, however, only one annotation of drinking. This is due, at least in part, to drinking being a short action or one that has been incorporated into a broader activity. This was noted by P2 during the interview: ‘*Those activities are quite detailed activities, but sometimes you do activities—like eating, like going to the kitchen and getting water – where it’s just like a 10 s or 20 s activity, it’s quite difficult to stop outside the kitchen and then do annotation, and then go in and then go out, and do the annotation and start the annotation again.*’

### 4.3. The Language of Labelling

**Figure 7:**
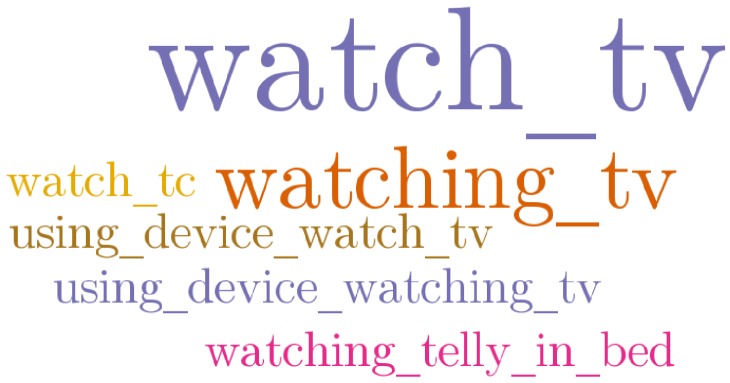
Tag cloud of various terms used for one concept (‘*watching TV*’).

It is common within the data set for various terms to be constructed and used for any given normalised term. Figure 7 is a tag cloud that visually summarises the various terms used for the activity of watching television. This is a weighted list where the term ‘*watch tv*’ is the largest as it was used the most (39 instances), followed by ‘*watching tv*’ that was used eight times; all other terms were used only once. As can be seen, two verb forms (the gerund and the infinitive) were used. Various structures were used, including the straightforward noun phrase (gerund + noun) or infinitive phrasal forms, without preposition (infinitive+noun), hierarchical structures (‘*using device: watching TV*’) and a lengthier fragment employing a colloquialism (‘*telly*’). Ten participants employed the infinitive phrasal form, two participants repeatedly employed the noun phrase, a single participant employed two variants of a hierarchical structure and one employed the colloquial term ‘telly’ using the Voice modality. One orthographic error (‘*watch_tc*’) demonstrates a further cause for misinterpretation or unusable annotations where free-text input is used.

Reviewing the distribution of term use for a particular kind of vocabulary—specifically, use of terms in describing meal preparation—shows a surprisingly broad distribution of usage (see Figure 6). The term ‘*breakfast*’ is used for any point before midday, whilst the term ‘*dinner*’ is used at any point after around 2:00 p.m. The term ‘*lunch*’ is used at a low rate throughout the day. In reviewing the terms used here, caveats apply. Firstly, a proportion of participants are not native English speakers; secondly, the sample size is not very large; thirdly, participants were often absent from the home throughout the day, although they may have prepared a daytime meal to take with them. Nonetheless, the data suggest a significant overlap in the usage of these terms—they appear to be used interchangeably in some circumstances.

#### Performative Usage

A small subset of the terms used could not be fully normalised against the SPHERE ontology. These fall into three broad categories: transition-related annotations (i.e., ‘*leaving the house*’ and ‘*getting ready to leave*’, ‘*entering house*’—only a small number of transitions and states relating to preparation for transitions are currently encoded in the SPHERE ontology), annotations related to events that occur outside the home (i.e., ‘*pub*’ or ‘*shopping*’) and idiosyncratic terms that are more difficult to categorise. In particular, a few of these idiosyncratic terms may be categorisable as performative in nature; for example, one participant’s repeated use of the term ‘*hand stands!*’, an exclamation mark included in each case, suggests an element of play. The action of completing a hand stand may be completed in the awareness that it is an unusual activity, that it will be unusual or unique in the data set. Another performative usage of the system is the term ‘*ahahaha*’, input at the end of a stay, presumably at the time of the participant’s departure. Such annotations, unlike the other two subsets of un-normalisable terms, are unlikely to be directly useful for most studies on the basis of the data. This use of the system suggests that these actions are informed by an awareness of the researchers who will be using the data.

### 4.4. Duration of Activities

The duration of activities measured through the app (Table 3) in combination with interview data emphasises just how difficult some participants found it to annotate their activities in real time. As an example, P1 had a relatively high number of activities lasting under a minute including getting dressed and reading; this participant reported finding the annotation process impractical and expressed a desire to be able to manually edit start times for activities. Similarly, other participants mentioned forgetting to log activities—especially spontaneous activities that were interleaved with planned activities—and recording them after the fact. To achieve this, they either started and stopped logging an activity almost instantly, like P1 did; alternatively, some participants started logging an activity after it had been completed but waited an amount of time they judged to be similar to length of the activity before stopping it. The latter approach was thus described by P3: ‘*it’s precise but it’s not at all accurate*’.

Another limitation of measuring the duration of activities through the app was that participants felt several activities do not have a clear beginning and end. One example given was the cup of coffee the participant had over the course of the interview, which made them wonder whether they had several short instances of drinking or one long activity that started at the first sip and finished when the last drop was consumed. Overall, this suggests that labelling acquired through self-annotation may not always align with sensor data but rather may serve as a flag for an activity has occurred within a short time frame preceding the annotation.

### 4.5. Location

Location information was gathered alongside activity data. However, as explained in Section 3.2, not every mode of logging carried this information. For example, when the ‘*Ongoing activities*’ list or ‘*Finish all*’ options were used to terminate activities started with NFC or the room-based list, this generally resulted in the lack of location information for the end of that activity. While the app allows users to enter this information manually via the ‘*My history*’ list of terminated activities, participants did not tend to do this. This is seen in Table 4 where the ratio concerning how many logs carried the start location and the latter concerning the same for end location are tabulated. None of the entries in the table has the second number higher than the first one. This suggests there may be a trade-off between modes to start and stop logging activities that are convenient for the user and the completeness of location data. Even when location is recorded, it is foreseeable that this information may not always match up with the data recorded by the sensors—for example, one participant explained that they logged ‘*cooking*’ with kitchen as the pre-defined associated location when they were heating up a pizza in the oven, yet they spent most of the duration of this activity in the living room.

The second to last column in Table 4 captures occurrences of entries that do not have location information, both for the start location (left side) and for the end location (right side). Only 17 out of 677 logs, corresponding to 100% of voice entries, did not carry the start location. The figure for end location was significantly larger at 224 instances, which is a just over 3% of the total number of entries. The fact that participants did not enter location manually and overall did not mention location during the interviews suggests that they were not particularly motivated to record this information. Only one participant discussed their preoccupation with recording location and cited this as one reason to use the room-based list over other modes of logging. For 11 instances of unterminated activities, these only contained information relating to where the activity was started but not where it was terminated. This may partly be explained by the ‘*Export All*’ feature, which was used at the end of each stay to force all the information that lives in the cache of the app to be immediately pushed to the server.

## 5. Discussion

This paper builds on previous work [15] by expanding the study sample from three people in the earlier work to 12 people in the current work, living in a smart home for periods of two or three nights. These participants were provided with an app to annotate their activities and location, which comprised three different modes of logging as well as the option to manually edit entries. In doing so, the study aimed to understand people’s experience of self-annotation in a relatively naturalistic setting. Allowing participants to have control over the annotation of their own data accords with recent research [17,18], which has highlighted the importance of generating interpretations of smart home data that are situated, appropriate, and acceptable. This research is also in line with a move towards more naturalistic data sets (exemplified by [13]), an area ripe for research given the proliferation of domain adaptation [25] in machine learning. It is therefore important to gain a rich understanding of self-annotation practices, in order to encourage participant-led labelling that is meaningful and useful for all stakeholders in this process.

Self-annotation proved interesting to some participants in this study—for example, as a means of examining their everyday use of time. This resonates with other examples in the literature where people successfully self-annotate selected information about their everyday lives and activities, for various purposes such as time management, healthcare, or to support personal goals [32,34]. For this reason, it is likely to be more effective to place annotation activity in the context of users’ interests and wants. Additionally, where possible, it is preferable to encapsulate annotation activity into a broader activity that carries a benefit to the user and has a clearly articulated purpose. We found that logged time and duration of activities may not match up accurately with when and for how long they were actually performed. This was sometimes due to participants actively choosing to record the activity afterwards for their own convenience, as has been observed in other research [47]. We believe that supporting post hoc self-annotation is likely to increase data counts, although it may come at the cost of accuracy in timing. System designers should acknowledge at an early stage that repeated self-interruption for annotation comes at a significant cost (including postcompletion errors associated with increased cognitive load [44]) and that, consequentially, users may often be unwilling or unable to engage at this level. A system that enables the participant to identify some approximate time in the past and specify a post hoc annotation based on their estimation of timescales may therefore be beneficial. Whilst the expectation is that annotations collected in this way will be imprecise by comparison to contemporaneous annotation, they may still be useful, particularly for classifier validation purposes. A further possible drawback of such a post hoc annotation process may be a decrease in users’ motivation to annotate as they experience a build-up of unannotated activities. It is then worth investigating a flexible approach that allows users to annotate both in real time and post hoc, which could leverage aspects of self-annotation that participants found engaging such as facilitating self-reflection.

Another challenge raised by self-annotation in our study was acquiring location information, either accurately (for activities correlated with a location but where participants were not present for the duration) or at all (particularly true for activities logged through voice). This suggests that participants were either not aware of the extra steps required to record location for the voice modality, or were not motivated to record this additional information. Further work is required to explore ways to better capture this type of information, perhaps through studies focused more on location rather than activities (e.g., [63]). The SPHERE platform currently uses the BLE technology to localise users within the house, based on received signal strength indicator (RSSI) from the wearable sensors to the stationary BLE-enabled gateways—the results of this research are yet to be reported. Such a (near) real-time localisation service could serve as an optional input to the app, either during its execution or upon data curation. Other solutions exist in this space, although this was not the primary focus of this study where we sought to collect timestamped activity labels. Localisation in the wild, as compared to the controlled laboratory setting, may be influenced by many contextual factors such as building architecture and materials. There is therefore a case for the evaluation of the annotations by sensor data, just as localisation data may be be validated by annotations in an iterative process.

Surprisingly, we observed a few cases where participants explored playful use of the app beyond a strict activity logging purpose—these included idiosyncratic terms, which can be categorised as performative in nature [64]. We argue that it may be advantageous to support this unique activity afforded by the multi-modality of the app. In particular, such an informal backchannel may function as a means of engagement with researchers and with the research process, thus encouraging user ‘buy-in’ and providing a motivation for ongoing participation.

Given the flexibility of the app in terms of logging and editing entries, as well as evidence in the literature of diverse interpretations of labels [57], we were interested in the language used for labelling activities. A common approach for exploration of terminology in use is to consider its distribution. Terminology in use tends to co-occur in ways that reflect the meaning of the terms used; in other words, the statistical distribution of terms reflect their meaning [65]. Consider the case of annotations within a home. There are certain terms that we would expect naively to co-occur because activities are linked or we understand that they are part of a sequence of events (e.g., ‘*brushing teeth*’ and ‘*going to bed*’; ‘*preparing a drink*’ and ‘*drinking*’). However, in our study, one label was used often at the exclusion of its associated pair. We also observed use of diverse terminology, including colloquialisms, even within a single participant’s data set. Hence, systems that mirror individual perception and practices to some level—that is, systems that take into account users’ perceptions, practices and terminology or classification practices—may be preferable to systems that enforce a formal classification system with which the user may be less familiar. By allowing users to input their own labels, a comprehensive graph representing such annotations could be drawn in order to contribute to the formalisation of ontology terms and concepts over time. Such terms can then be added into the ontology or replace already existing ones. As cohort size grows and diversifies, a folksonomic (social-tagging) approach to vocabulary development can be employed alongside the traditional ontology and formal labelling system already employed in this work. These findings have implications for the ontology [60,62] underlying much of this data collection. The concept spine (the ontology or taxonomy) does not need to closely resemble the user interface or the labels shown to or provided by users. It is worth considering mapping user-friendly terminology to the concept spine. The use of this information for ontology development and validation is discussed further elsewhere [66].

Some strengths and limitations need to be considered when interpreting the results of this work. While we do not claim that the experiences of the participants are generalizable to the wider population, we argue that the rich data combined with our rigorous approach to analysis contributed to credible findings that are worthy of further work in order to develop successful annotation tools that can be deployed in the real world. Embedding this research within stays in a smart home lasting a few days enabled participants to settle into their own routines and, as such, generated fairly naturalistic data. Although the process of logging one’s own activities disrupts the fluidity of daily life, other forms of manual annotation carry their drawbacks in terms of scalability and their own risks in terms of modified behaviour due to the presence of an annotator [13,14] or video-recording of participants [12]. Self-annotation approaches thus have the potential to play an important role as pervasive technologies make their way from the lab into people’s homes.

## 6. Conclusions and Future Work

In seeking to understand self-annotation from the user’s perspective, this research has uncovered a number of avenues for further work. Firstly, there is work to be done to confront self-annotations against sensor-derived data. Some entries, especially those terminated via ‘*Ongoing list*’ or ‘*Finish all*’ and those entered via voice, do not carry location information. The localisation feature of the SPHERE system may provide a valuable source of data, which could add knowledge about the accuracy of self-reported data in terms of delay in reporting and duration of activities. In future iterations of the SPHERE system, replacement of existing WiFi access points with systems implementing upcoming standards such as 802.11mc could provide additional localisation data with potential improvements in accuracy and reliability, further enhancing the annotation data set. Secondly, a clear next step would be a study involving a greater number of participants and conducted in their own homes, equipped with the SPHERE system. It is anticipated that this would further diversify the sample in several key aspects, including familiarity with technology, health status, cultural background, and others. This might contribute to growing the list of terminology in use, which could in turn inform a more user-centred ontology as well as permitting the development of a folksonomic knowledge graph. Lastly, moving on from NFC, there is room for research themes such as ‘smart objects’ that contain or are readily monitored by sensors and which can therefore be used, mindfully or otherwise, as annotation sources for a smart home. Here, there is potential for a significant design element to create smart objects that are not only functional but also desirable, in order to encourage annotation through interacting with them.

## Figures and Tables

**Table 1:** Modes of logging and their features/limitations.

Mode	Start Activity	Terminate Activity	Location Info	Room
Room-based list	✓	✓	✓	All
NFC	✓	✓	✓	Kitchen, bedroom(s)
Voice	✓	✗	Not explicitly	All
Ongoing list	✗	✓	✗	All
Finish all	✗	✓	✗	All

**Table 2:** Modes of logging (RB = Room-based list; UT = unterminated; DA = Average number of activities logged per day, rounded to the nearest integer).

P. ID	START			END					DA
	RB List	**NFC**	**Voice**	**RB List**	**Ongoing List**	**Finish All**	**NFC**	**UT**	
P1	118	—	7	74	30	17	—	4	31
P2	72	—	—	45	27	—	—	—	24
P3	54	14	1	51	7	6	5	—	35
P4	26	31	2	20	17	—	22	—	30
P5	47	22	—	2	54	—	13	—	23
P7	59	—	—	37	13	5	—	4	20
P8	28	8	—	16	18	—	—	2	18
P9	43	—	—	30	13	—	—	—	14
P10	64	—	—	59	5	—	—	—	21
P12	74	—	7	68	12	—	—	1	27
**Avg**	58.5	18.75 (7.5)	4.25 (1.7)	40.2	19.6	9.3 (2.8)	13.3 (4)	2.75 (1.1)	24

**Table 3:** Duration of logged activities (Unk. = Unknown).

P. ID	<1 min	1–3 min	3–5 min	<10 min	<30 min	<1 h	1–2 h	>2 h	Unk.
P1	46	14	13	8	19	9	3	9	4
P2	10	5	9	15	10	7	7	9	—
P3	22	8	7	8	14	3	3	4	—
P4	29	5	3	4	10	2	2	4	—
P5	3	6	10	14	13	4	6	13	—
P7	4	11	4	9	10	8	6	3	4
P8	8	6	4	6	5	1	—	4	2
P9	4	1	6	5	12	9	3	3	—
P10	4	3	5	12	24	10	3	3	—
P12	14	10	10	20	14	6	3	3	1
**Total**	144	69	71	101	131	59	36	55	11

**Table 4:** Logged location (each entry contains (unsimplified) ratios on ‘Start Location’:‘End Location’; Hall = Hallway, Bed 1 = Bedroom 1, Bed 2 = Bedroom 2, LR = Living Room, Out = Outside, ? = Not recorded, UT = unterminated).

P. ID	Bathroom	Bed 1	Bed 2	Hall	Kitchen	LR	Out	Study	Toilet	?	UT
P1	8:5	11:11	19:7	6:5	42:29	10:6	5:2	10:2	7:7	7:47	4
P2	3:2	14:14	7:-	1:1	16:8	15:9	2:1	1:1	13:9	-:27	-
P3	1:1	11:8	-:-	4:4	27:20	19:19	-:-	4:2	2:2	1:13	-
P4	-:-	1:-	12:5	4:4	25:22	15:11	-:-	-:-	-:-	2:17	-
P5	5:-	9:1	1:-	-:-	22:13	27:1	5:-	-:-	-:-	-:54	
P7	4:3	-:-	6:4	-:-	15:15	20:9	4:-	4:-	6:6	-:18	4
P8	2:1	4:3	-:-	-:-	14:5	13:5	-:-	-:-	3:2	-:18	2
P9	1:1	11:6	-:-	-:-	11:11	14:8	-:-	-:-	6:4	-:13	-
P10	1:-	16:16	-:-	-:-	23:22	24:21	-:-	-:-	-:-	-:5	-
P12	1:1	27:23	-:-	5:5	24:24	8:6	-:-	-:-	9:9	7:12	1
**Total**	26:14	104:82	45:16	20:19	219:169	165:95	16:3	19:5	46:39	17:224	11

## Supplementary Materials

The data set collected during this study and the SPHERE ontology of daily living are available online via the https://data.bris.ac.uk/ data repository: doi:10.5523/bris.1234ym4ulx3r11i2z5b13g93n7 and doi:10.5523/bris.23uy4kg0al1kt2qye1y2athlr1 respectively.

## Abbreviations

AR	Activity Recognition
ADL	Activities of Daily Living
ATM	Automated Teller Machine
ARDUOUS	International Workshop on Annotation of useR Data for UbiquitoUs Systems
BLE	Bluetooth Low Energy
CSV	Comma Separated Values
JSON	JavaScript Object Notation
HCI	Human-Computer Interaction
MAC (address)	Media Access Control address
NFC	Near Field Communication
PIM	Personal Information Management
RSSI	relative Received Signal Strength Indicator
SPHERE	Sensor Platform for HEalthcare in a Residential Environment
UI	User Interface
